# Absence of KRAS Mutation as an Indicator of Pancreatic Metastasis Originating From Lung Cancer: A Case Report

**DOI:** 10.7759/cureus.85759

**Published:** 2025-06-11

**Authors:** Shuhei Suzuki, Soshi Oyama, Takanobu Kabasawa, Hidenori Sato, Hidekazu Matsumoto

**Affiliations:** 1 Department of Internal Medicine, Yamagata Prefectural Shinjo Hospital, Yamagata, JPN; 2 Department of Pathology, Yamagata University, Yamagata, JPN; 3 Department of Surgery, Yamagata University, Yamagata, JPN; 4 Department of Surgery, Yamagata Prefectural Shinjo Hospital, Yamagata, JPN

**Keywords:** cancer genomics, egfr mutations, kras mutation, non-small cell lung cancer, pancreas tumor

## Abstract

Differentiating between primary pancreatic adenocarcinoma with pulmonary metastasis and primary lung adenocarcinoma with pancreatic metastasis presents a significant diagnostic challenge due to histological similarities. This distinction is crucial as it directly impacts treatment strategies and patient outcomes. We present a case of a 74-year-old female with concurrent pancreatic and pulmonary masses discovered during a routine health screening. Initial evaluations suggested primary pancreatic cancer with pulmonary metastasis based on imaging characteristics. However, molecular analysis revealed the absence of *KRAS* mutation in the pancreatic lesion, which is highly unusual for primary pancreatic adenocarcinoma. Further immunohistochemical studies showed thyroid transcription factor-1 (TTF-1) positivity, and genetic testing identified an* EGFR* exon 19 deletion (E746_A750del), confirming the diagnosis of metastatic lung adenocarcinoma to the pancreas. This case highlights the critical importance of molecular profiling in distinguishing between primary and metastatic lesions when conventional diagnostic methods are inconclusive. The absence of *KRAS* mutation in a pancreatic adenocarcinoma should prompt consideration of metastatic disease, particularly from the lung. This case illustrates that comprehensive molecular and immunohistochemical analyses can prevent misdiagnosis and ensure appropriate targeted therapy selection in cases with diagnostic uncertainty.

## Introduction

Pancreatic cancer remains one of the most lethal malignancies [1,2]. The majority of pancreatic cancers are ductal adenocarcinomas, characterized by their aggressive behavior and late presentation [3]. Conversely, lung cancer is the leading cause of cancer-related deaths worldwide, with adenocarcinoma being the most common histological subtype [4]. The diagnostic dilemma arises when patients present with both pancreatic and pulmonary lesions, as determining the primary site becomes challenging yet critical for appropriate treatment planning. This difficulty is particularly pronounced when both lesions are adenocarcinomas, as the histological features may appear remarkably similar under conventional microscopic examination. While pancreatic adenocarcinoma commonly spreads to the lungs in advanced disease, the reverse scenario - lung cancer metastasizing to the pancreas - represents a rare occurrence among all pancreatic metastases [5].

Molecular profiling has emerged as a valuable tool for resolving such diagnostic challenges. *KRAS* mutations are nearly ubiquitous in pancreatic ductal adenocarcinoma, with a prevalence exceeding 85% [2]. Conversely, *KRAS* mutations are present in only approximately 30% of lung adenocarcinomas [6], while *EGFR* mutations occur in 10-15% of non-Asian and up to 50% of Asian patients with non-small cell lung cancer (NSCLC) [7].

Immunohistochemical markers such as thyroid transcription factor-1 (TTF-1) and napsin A are typically positive in lung adenocarcinomas but negative in pancreatic adenocarcinomas [8]. However, these markers are not always conclusive, and molecular analysis provides additional diagnostic precision.

Here, we present a case of a 74-year-old female with concurrent pancreatic and pulmonary masses, where the absence of *KRAS* mutation in the pancreatic lesion served as a critical clue leading to the diagnosis of metastatic lung adenocarcinoma to the pancreas. The subsequent identification of an *EGFR* exon 19 deletion guided the selection of appropriate targeted therapy. This case underscores the importance of comprehensive molecular profiling in distinguishing primary from metastatic lesions in complex oncological scenarios.

## Case presentation

A 74-year-old female with a smoking history of 17 cigarettes daily for 30 years was referred to our gastroenterology department after a pancreatic mass was incidentally detected during a routine health screening. Her medical history included endometrial cancer (age 66, T1bN0M0, endometrioid type, treated with total abdominal hysterectomy, bilateral salpingo-oophorectomy, pelvic lymph node dissection, and six cycles of docetaxel plus carboplatin), thymoma (age 60, type B3, treated with extended thymectomy via median sternotomy with right lung and partial pericardial resection), and hypertension. She had no known allergies, reported occasional alcohol consumption, and worked in sales. Her family history was notable for pancreatic cancer in a maternal cousin in his 90s. Her only medication was amlodipine 5 mg daily.

**Figure 1:**
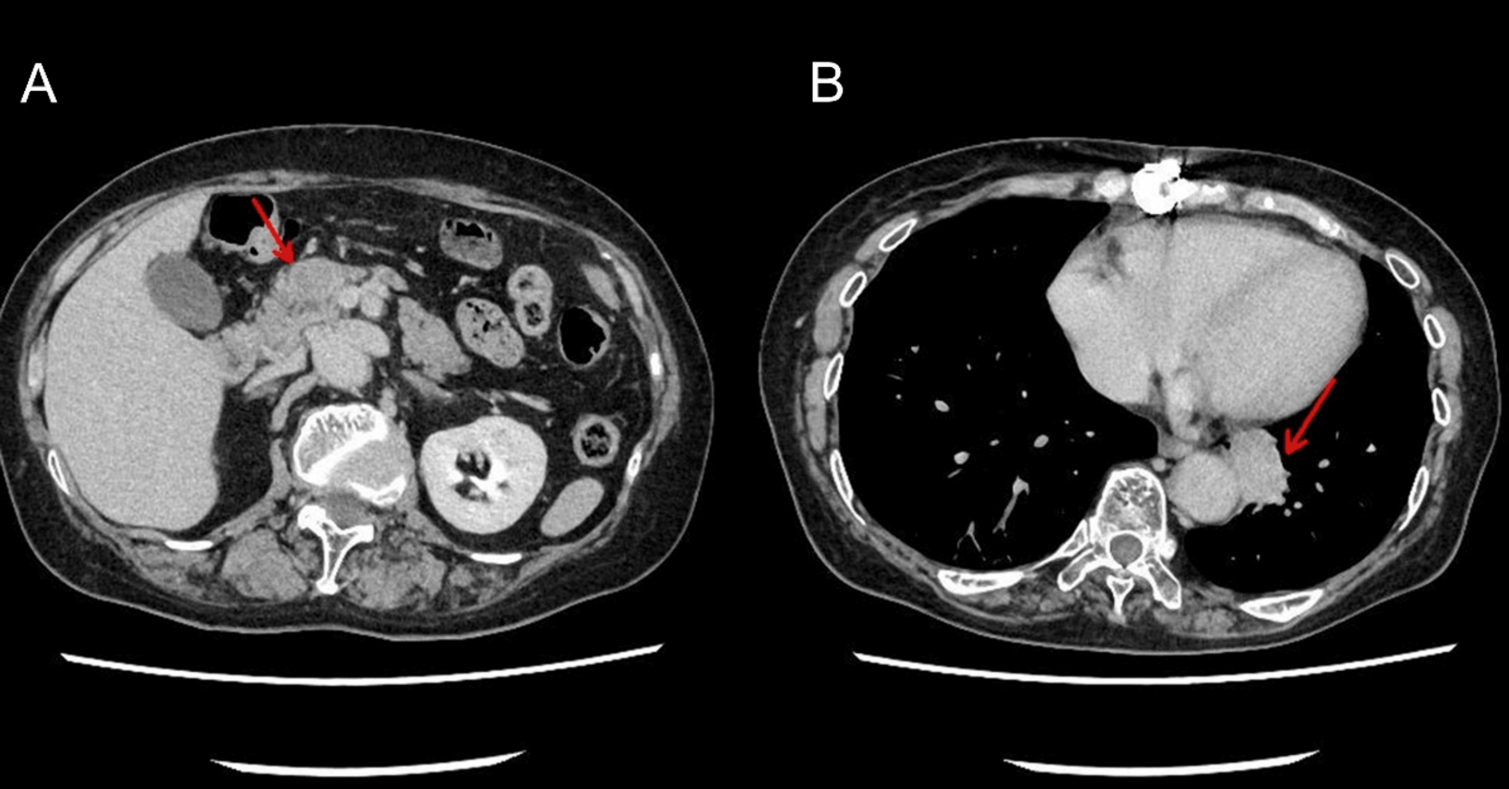
Contrast-enhanced computed tomography images. (A) Axial image showing a 20-mm mass protruding from the ventral aspect of the pancreatic head (arrow). (B) Axial image demonstrating a lobulated mass in the left lower lobe of the lung adjacent to the aorta (arrow).

Abdominal contrast-enhanced computed tomography (CT) revealed a 20-mm mass protruding from the ventral aspect of the pancreatic head with slight hypoenhancement in all phases compared to the pancreatic parenchyma (Figure 1A). There was no distal pancreatic atrophy or main pancreatic duct dilation. Additionally, a lobulated mass lesion was identified in the left lower lobe of the lung adjacent to the aorta (Figure 1B).

**Figure 2:**
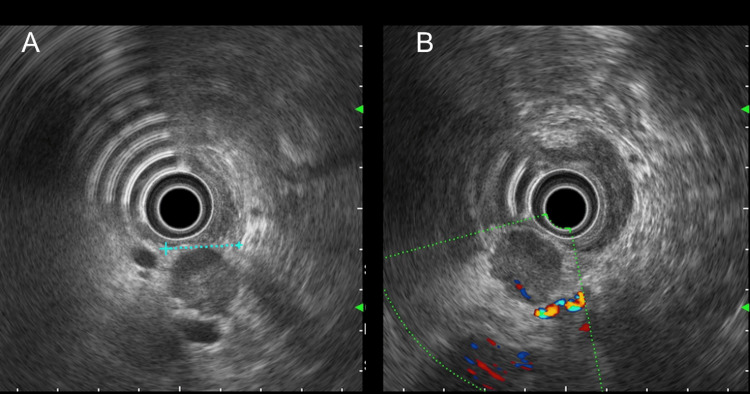
Endoscopic ultrasound images of the pancreatic mass. (A) B-mode image showing a well-defined 18-mm hypoechoic mass in the pancreatic head. (B) Doppler image demonstrating peripheral blood flow within the mass.

Pancreatic magnetic resonance imaging (MRI) showed a 20-mm mass bulging from the pancreatic head, appearing hypointense on T1-weighted images, heterogeneously hypointense to hyperintense on T2-weighted and short tau inversion recovery (STIR) images, with ring-like high to low intensity signals predominantly at the periphery on diffusion-weighted imaging (DWI) and apparent diffusion coefficient (ADC) mapping. Dynamic studies demonstrated heterogeneous enhancement predominantly at the periphery from the arterial to equilibrium phases. The main pancreatic duct remained patent without dilation. Endoscopic ultrasound (EUS) at another hospital revealed a well-defined 18-mm hypoechoic mass in the pancreatic head (Figure 2A). Doppler imaging showed peripheral blood flow (Figure 2B). EUS-guided fine-needle aspiration (EUS-FNA) yielded moderately differentiated adenocarcinoma. 

**Figure 3:**
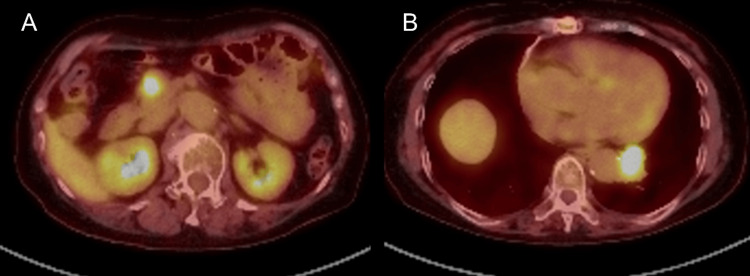
Positron emission tomography/computed tomography images. (A) High fluorodeoxyglucose (FDG) uptake observed in the pancreatic head mass (standardized uptake value maximum (SUVmax): early phase 7.62, delayed phase 9.41). (B) High FDG uptake observed in the left lower lobe lung mass (SUVmax: early phase 8.75, delayed phase 10.48).

At our institution, two attempts at bronchoscopy for the pulmonary lesion were non-diagnostic. Positron emission tomography/computed tomography (PET/CT) revealed high uptake in both the pancreatic head tumor (standardized uptake value maximum (SUVmax): early phase 7.62, delayed phase 9.41) and lung tumor (SUVmax: early phase 8.75, delayed phase 10.48) with no other areas of abnormal uptake (Figures 3A, 3B).

**Figure 4:**
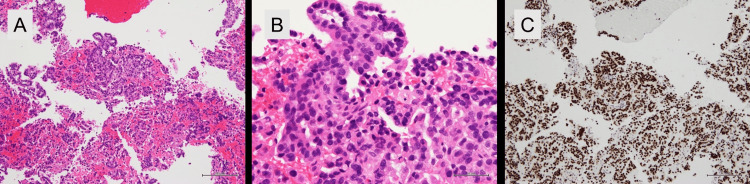
Histopathological examination of the pancreatic mass. (A) Hematoxylin and eosin (H&E) staining (×100) showing adenocarcinoma infiltration. (B) H&E staining (×400) demonstrating moderately differentiated adenocarcinoma with glandular formation. (C) Positive immunohistochemical staining for thyroid transcription factor-1 (×100), supporting lung origin.

Laboratory findings included serum IgG4 of 12.3 mg/dL (normal range). Tumor markers were unremarkable: carcinoembryonic antigen (CEA) 3.780 ng/mL, carbohydrate antigen 19-9 (CA19-9) 8.03 U/mL, squamous cell carcinoma antigen (SCC) 0.6 ng/mL, neuron-specific enolase (NSE) 11.5 ng/mL, pro-gastrin-releasing peptide (ProGRP) 64.2 pg/mL, cytokeratin 19 fragment (CYFRA) below measurable limits, soluble interleukin-2 receptor (sIL-2R) 282 U/mL, duke pancreatic monoclonal antigen type 2 (DUPAN-2) below measurable limits, s-pancreas antigen-1 (SPAN-1) 7.5 U/mL, and cancer antigen 125 (CA125) 16.70 U/mL. The case was presented at our Cancer Treatment Board, but differential diagnosis between pancreatic cancer with lung metastasis, synchronous primary lung and pancreatic cancers, or lung cancer with pancreatic metastasis remained unresolved, preventing a definitive treatment plan. A breakthrough occurred when molecular analysis of the pancreatic FNA specimen revealed the absence of the *KRAS* mutation. *KRAS* mutation analysis was performed using the polymerase chain reaction-reverse sequence-specific oligonucleotide (PCR-rSSO) method, which confirmed wild-type *KRAS *status. Given that most pancreatic ductal adenocarcinomas harbor *KRAS *mutations, this finding raised suspicion for metastatic disease. Subsequent immunohistochemical staining revealed TTF-1 positivity in the pancreatic specimen (Figures 4A-4C), and subsequent multi-gene testing using the AmoyDx® Lung Cancer Multi-Gene PCR Panel (Amoy Diagnostics Co., Ltd., Xiamen, China) identified an *EGFR* exon 19 deletion (E746_A750del), confirming the diagnosis of *EGFR*-mutated lung adenocarcinoma with pancreatic metastasis.

Based on these findings, the patient was initiated on combination therapy with osimertinib, carboplatin, and pemetrexed for metastatic *EGFR*-mutated lung adenocarcinoma.

## Discussion

This case highlights the critical role of molecular profiling in resolving diagnostic challenges when patients present with multiple lesions of similar histology. The initial presentation with concurrent pancreatic and pulmonary masses posed a significant diagnostic dilemma, which was ultimately resolved through molecular and immunohistochemical analyses.

The absence of *KRAS* mutation in the pancreatic lesion was the pivotal clue that prompted further investigation. *KRAS* mutations are present in over 85% of pancreatic ductal adenocarcinomas [2], making their absence highly unusual for primary pancreatic cancer. Conversely, *KRAS* mutations occur in only about 30% of lung adenocarcinomas [4]. This molecular discordance served as a crucial indicator that the pancreatic mass might not represent a primary lesion. In clinical practice, such molecular inconsistencies with expected tumor profiles should prompt consideration of alternative diagnoses, including metastatic disease. While *EGFR* mutations are rarely observed in primary pancreatic adenocarcinomas, they are well-established oncogenic drivers in lung adenocarcinomas, particularly in Asian populations and non-smokers. The combination of *KRAS*-wildtype status and *EGFR* mutation positivity in a pancreatic lesion strongly suggests metastatic origin rather than primary pancreatic malignancy.

The subsequent positive TTF-1 immunohistochemistry further supported lung origin, as TTF-1 is expressed in approximately 85% of lung adenocarcinomas but is typically negative in pancreatic adenocarcinomas [9]. The detection of an *EGFR* exon 19 deletion provided additional supporting evidence for lung origin. *EGFR* mutations are generally associated with a subset of lung adenocarcinomas, particularly in East Asian populations, non-smokers, and females, while such mutations are not typically observed in primary pancreatic malignancies. This case demonstrates several challenges in diagnosing metastatic disease when both primary and metastatic lesions are adenocarcinomas. First, histological features alone are often insufficient for differentiation. Second, imaging characteristics may not clearly distinguish between primary and metastatic lesions. In our case, both the pancreatic and pulmonary masses demonstrated similar enhancement patterns and FDG avidity on PET/CT, providing no clear distinction between primary and metastatic disease. SUV values reflect metabolic activity and tumor aggressiveness rather than origin, and histologically similar lesions (adenocarcinomas) often exhibit comparable glucose uptake regardless of whether they represent primary or metastatic disease.

Furthermore, obtaining diagnostic tissue from lung lesions can be challenging, as evidenced by our two non-diagnostic bronchoscopy attempts. In such scenarios, molecular analysis of more accessible lesions, such as the pancreatic mass in our case, might become invaluable. Targeted molecular approaches suitable for small FNA specimens can provide crucial diagnostic information when tissue sampling from other sites proves challenging.

The identification of an actionable *EGFR* mutation had immediate therapeutic implications. *EGFR*-targeted therapies have demonstrated superior efficacy compared to conventional chemotherapy in* EGFR*-mutated NSCLC [10]. Our patient was started on osimertinib, a third-generation *EGFR* tyrosine kinase inhibitor, in combination with carboplatin and pemetrexed [11].

To better understand the landscape of pancreatic metastases from lung cancer in Japan, we analyzed data from the Center for Cancer Genomics and Advanced Therapeutics (C-CAT) database [12]. As of April 16, 2025, among 82,773 registered cases, 5,779 were lung cancers, of which 4,586 were NSCLC (2,829 males, 1,757 females, with the majority in their 70s at 1,582 cases). Pancreatic metastases were documented in 75 NSCLC cases, with only 10 having *EGFR*-mutated adenocarcinoma (Table 1). Notably, all cases with pancreatic metastases also had metastases to other organs, making our case of isolated pancreatic metastasis particularly unusual.

**Table 1:** Summary of EGFR-mutated lung adenocarcinoma cases with pancreatic metastasis registered in the Center for Cancer Genomics and Advanced Therapeutics database as of April 16, 2025. F1L: FoundationOne Liquid; F1: FoundationOne; TOP: GenMineTOP; F: Female; M: Male

Age	Sex	*EGFR* mutation	Metastatic site	Testing panel
86	M	L858R	Bone	F1L
51	F	L858R, C797S	Brain, Liver, Lymph Node	TOP
69	F	E709K, G719A, D368N	Brain, Lung, Bone, Lymph Node	F1L
72	F	C775_R776insPHVC	Bone, Pleura, Lymph Node	F1
71	M	E746_A750del, T790M	Pleura, Liver, Lymph Node	F1L
60	F	L858R, D301H, L718Q	Brain, Lung, Liver, Adrenal Gland, Kidney, Bone	F1L
41	F	E746_A750del, T790M	Brain, Lung, Pleura, Liver, Bone	F1L
69	M	G719C, D761Y, S306L	Lung	F1L
62	M	L747_A750delinsP, L718W, C797D	Lung	F1
72	F	E746_A750del	Lymph Node, Adrenal Gland	F1

Pancreatic metastases from lung cancer generally portend a challenging clinical course, with many patients facing limited survival periods after diagnosis. However, patients with *EGFR*-mutated NSCLC may experience better outcomes due to the availability of effective targeted therapies [10].

This case emphasizes that when conventional diagnostic methods are inconclusive, molecular profiling should be considered to distinguish between histologically similar lesions. The absence of characteristic mutations, such as *KRAS,* in a presumed pancreatic adenocarcinoma should prompt consideration of metastatic disease and trigger additional molecular and immunohistochemical studies.

## Conclusions

This case illustrates the value of molecular profiling in resolving diagnostic challenges when patients present with multiple adenocarcinomas of uncertain origin. The absence of *KRAS* mutation in a pancreatic adenocarcinoma served as a critical clue suggesting metastatic disease rather than a primary pancreatic malignancy. Subsequent identification of TTF-1 positivity and an *EGFR *exon 19 deletion confirmed the diagnosis of metastatic lung adenocarcinoma to the pancreas, enabling appropriate targeted therapy. Our experience highlights the importance of considering molecular discordance when evaluating patients with multiple lesions and demonstrates how targeted molecular testing can guide appropriate diagnosis in complex oncological cases. Clinicians should maintain a high index of suspicion for metastatic disease when molecular findings do not align with the expected profile of presumed primary malignancies.

As precision oncology continues to evolve, molecular profiling will likely play an increasingly important role in diagnosis, particularly in cases where conventional approaches are inconclusive. This case adds to the limited literature on isolated pancreatic metastases from lung cancer and emphasizes the value of multidisciplinary collaboration in managing diagnostically challenging cases.
